# Effects of Insufficient Physical Activity on Mortality and Life Expectancy in Jiangxi Province of China, 2007-2010

**DOI:** 10.1371/journal.pone.0109826

**Published:** 2014-10-14

**Authors:** Gang Xu, Xuemei Sui, Shiwei Liu, Jie Liu, Junxiu Liu, Yichong Li, Shouqing Huang, Zhengzhen Wang, Steven N. Blair

**Affiliations:** 1 Department of Preventive Medicine, School of Basic Medicine, Jiangxi University of Traditional Chinese Medicine, Nanchang, Jiangxi, China; 2 Department of Exercise Science, Arnold School of Public Health, University of South Carolina, Columbia, South Carolina, United States of America; 3 National Center for Chronic and Noncommunicable Disease Control and Prevention, Chinese Center for Disease Control and Prevention, Beijing, China; 4 Division of Chronic Disease Control and Prevention, Jiangxi Province Center for Disease Control and Prevention, Nanchang, Jiangxi, China; 5 Department of Epidemiology and Biostatisitcs, Arnold School of Public Health, University of South Carolina, Columbia, South Carolina, United States of America; 6 The Second People's Hospital Affiliated to Fujian University of Traditional Chinese Medicine, Fuzhou, Fujian, China; 7 Division of Exercise Rehabilitation, Beijing Sport University Sport Rehabilitation College, Beijing, China; IPO, Inst Port Oncology, Portugal

## Abstract

**Background:**

Physical inactivity remains an under-researched field in terms of studying burden of disease at provincial level, and no studies have examined the effects of inactivity on life expectancy (LE) in China. The purpose of this study was to estimate mortality risk and LE effects associated with insufficient levels of physical activity in Jiangxi province.

**Methods/Findings:**

Prevalence of risk factors and mortality counts were extracted from Chronic Diseases and Risk Factors Surveillance Survey (CDRFSS) and Disease Surveillance Points system (DSP), respectively. Insufficient physical activity (IPA) was defined as less than 150 minutes of moderate-intensity physical activity or 60 minutes of vigorous-intensity physical activity per week, accumulated across work, home, transport and discretionary domains. Population-attributable fractions (PAF) were used to calculate the mortality attributable to risk factors, and life table methods were used to estimate the LE gains and LE shifts. Monte Carlo simulation techniques were used for uncertainty analysis. Overall, 5 885 (95% uncertainly interval (UI), 5 047–6 506) and 8 578 (95% UI, 8 227–9 789) deaths in Jiangxi province were attributable to IPA in 2007 and 2010, respectively. The LE gains for elimination of attributable deaths were 0.68 (95% UI, 0.61–076) in 2007, and increased to 0.91 (95% UI, 0.81–1.10) in 2010. If the prevalence of IPA in 2010 had been decreased by 50% or 30%, 3 678 (95% UI, 3 220–4 229) or 2 090 (95% UI, 1 771–2 533) deaths would be avoided, and 0.40 (95% UI, 0.34–0.53) or 0.23 (95% UI, 0.16–0.31) years of LE gained, respectively.

**Conclusions:**

Adults in Jiangxi province of China have a high and increasing prevalence of IPA. Due to the deaths and potential LE gains associated with IPA, there is an urgent need to promote physical activity, one of the most modifiable risk factors, within China's health care reform agenda.

## Introduction

Physical inactivity has been associated with higher risk of many chronic diseases, including coronary heart disease [1], [2], ischemic stroke [3], [4], type 2 diabetes [5], [6], colon cancer [7] and breast cancer [8]. Physical inactivity also is linked to lower life expectancy (LE) [9]–[12]. Conversely, higher levels of physical activity are associated with lower risk of mortality and longer LE [13]. The comparative risk assessment of the Global Burden of Disease Study indicated that insufficient physical activity (IPA) accounted for 3.2 million deaths and 2.8% of disability-adjusted life years in 2010, and ranked tenth among the 20 leading risk factors in attributable burden of disease [14]. The study estimated that approximately 0.5 million deaths and 3.6% of disability-adjusted life years in China were attributable to IPA, and that IPA ranked tenth among the 67 risk factors examined [15]. Non-communicable diseases account for 85% of annual deaths and are the leading cause of death in China [16]. The 2010 Global Burden of Disease Study reported that the most significant behaviors related to non-communicable diseases in China included dietary risks, smoking, alcohol use, and physical inactivity [15]. Little research, however, has examined the role of physical inactivity on the burden of disease in China, especially at the provincial level. In addition, no studies have examined changes in LE attributable to physical inactivity in China.

The five-year plan for national economic and social development of China, developed in 2011[17], proposed to increase LE by 1 year, from 73.5 years in 2010 to 74.5 years in 2015, as one of 28 core indices. Since then, each provincial government has developed and implemented programs to improve LE in local populations by 1 year. In addition, China launched its new health care reform plan in 2009, and is periodically monitoring progress toward achieving the objectives of the plan [18], [19]. Quantitative and comparative studies on mortality attributable to risk factors and LE gains attributable to reducing risk factor are good indices to evaluate public health performance and guide health intervention and policy-making.

Average weekly physical activity among adults ages 18–55 in China decreased 32% between 1991 and 2006 [20]. The Chinese Chronic Diseases and Risk Factors Surveillance Survey (CDRFSS) reported in 2010 that only 11.9% of Chinese ages 18 and above participated in physical activity regularly (defined as ≥3 times per week, and ≥10 minutes every time for recreational physical activities) and that 83.8% did not participate in any physical activities [21]. Jiangxi province, located in southeast China, is situated 24°29′14″ – 30°04′41″ north latitude and 113°34′36″ – 118°28′58″ east longitude, and spans from the banks of the Yangtze River in the north into hillier areas in the south and east. In 2010, national census data showed that the population of Jiangxi province was approximately 44.57 million, with 72.06% of the population engaged in agriculture (nationwide: 70.13%). The urbanization rate was 43.75% (nationwide: 50.27%). The proportion of the population <15 years of age and ≧65 years of age was 18.88% and 7.60%, respectively (nationwide: 14.01% and 8.92%, respectively), in 2010. Generally, Jiangxi has a larger labor force population and lower urbanization than the country as a whole.

The purpose of this study was to estimate the mortality due to IPA in Jiangxi province among adults from 2007 to 2010, based on Chinese CDRFSS data and the corresponding years' mortality data from the Chinese Disease Surveillance Points (DSP) system, and to estimate the number of deaths that could have been averted by reducing this risk factor at different counterfactual scenarios. LE gains and LE shift were calculated using life-table methods.

## Methods

### Data Sources

#### Exposure data of Insufficient Physical Activity

Self-reported data on the prevalence of IPA among Jiangxi province adults 18 years of age or older were retrieved from Chinese CDRFSS 2007 and 2010, which were independently carried out in the surveillance points (one point corresponds to one county or district) of DSP system [21]–[26]. All participants were randomly selected by a multi-stage stratified random sampling method; the sampling procedure and sample size at the national level are described elsewhere [21]–[25]. There were 1 554 and 2 772 participants surveyed in Jiangxi province in 2007 and 2010, respectively. Data on moderate-intensity or vigorous-intensity physical activity, using questions based on the Global Physical Activity Questionnaire [27], were collected across four domains - work, domestic, transport and discretionary time. In order to make the samples more representative of the total province, the sample weighting [28] and age- and sex- specific structure adjustment on the sample population were based on the whole population across the province [29]. Because the Global Physical Activity Questionnaire may not measure physical activity reliably among persons ≧70 years, data from participants in the age groups 70–74, 75–79 and ≧80 were combined with age group 65–69, and the same prevalence values were utilized for them.

Consistent with the methodology of comparative risk assessment in the Global Burden of Disease Study [30], physical activity was treated as a categorical variable: (1) Physical inactivity (level 1): doing no or very little physical activity at work, at home, for transport or during discretionary time. (2) Low physical activity (level 2): doing some physical activity but less than 150 minutes of moderate-intensity physical activity or 60 minutes of vigorous-intensity physical activity per week, accumulated across work, home, transport or discretionary domains. (3) Physical activity (level 3): at least 150 minutes of moderate-intensity physical activity or 60 minutes of vigorous-intensity physical activity per week accumulated across work, home, transport or discretionary domains. Levels 1 and 2 were defined as IPA.

#### Mortality data

Mortality data for 2007 and 2010 by underlying cause, age, and sex were extracted from the DSP system [26], which includes 5 surveillance points in Jiangxi province. Completeness of reporting and accuracy of cause of death are the main problems for any surveillance system. Therefore, we adjusted all the mortality data using propensity score methods based on the underreporting survey data and the redistribution of garbage code of cause of death. In addition, we adjusted the age- and sex-specific structure of the sample using the whole provincial population [29] to make it representative of the whole province.

#### Population data

The population of surveillance points in 2010 was obtained directly from 2010 census data. The population data for 2007 was interpolated from the 2000 and 2010 censuses by adjusting for annual variation by assuming a constant growth rate over all years.

#### Health outcomes and the relative risk (*RR*)

In accordance with comparative risk assessment in the Global Burden of Disease Study [30], ischemic heart disease, ischemic stroke, breast cancer, colon cancer and type 2 diabetes were identified as health outcomes etiologically associated with IPA. The *RR* for each health outcome with adjustment for measurement errors was also selected from the comparative risk assessment [30]. Though many studies [31]–[33] show the relationship of physical activity to weight status, obesity is excluded from the health outcomes in this study, consistent with comparative risk assessment methodology.

All enrolled participants in the CDRFSS gave written and informed consent for participation. The National Health and Family Planning Commission (NHFPC, previously Ministry of Health) of China and the Ethics Committee of the Chinese Center for Disease Prevention and Control approved the implementation of CDRFSS and mortality surveillance through the DSP system. The records/information of participants was de-identified prior to analysis.

### Statistical Analysis

#### Premature mortality attributable to risk factor

In our analysis, population-attributable fractions (PAF) were calculated to estimate the amount of premature deaths attributable to IPA by comparing the current population exposure with the theoretical minimum distribution of 0% IPA. The formula used was:



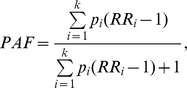
where *p_i_* is the prevalence of exposure level *i*, *RR_i_* is the *RR* of adverse health outcome in exposure level *i*, and *k* is the total number of exposure levels. For each disease causally associated with the risk factor, the attributable mortality was calculated by multiplying the PAF with the observed deaths by age and sex, then the attributable disease-specific mortality was summed as the whole mortality attributable to the risk factor [12]. If the counterfactual scenarios were not the theoretical minimum distribution of 0% IPA, but an alternative distribution with decreased prevalence of risk factor for interventions, the formula was:



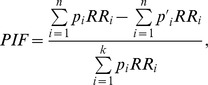
where PIF is potential impact fractions and *p_i_^'^* is the prevalence of exposure level *i* in alternative distribution.

#### Effects of risk factors on LE

We calculated the LE for each year using standard life table methods, based on the observed age-specific mortality (LEyt), as well as the alternative age-specific mortality by removing the deaths attributable to IPA (LEyr). The difference between the two sets of LEs at birth measures the LE gains (LEyr-LEyt). Also, LE shifts for the current distributions of risk factor exposure changed to an alternative scenario can be calculated using the same methods.

#### Alternative risk factor exposure distributions

We tracked the effects of IPA on mortality and LE in Jiangxi province relative to three different counterfactual exposure distributions, comparing with that in 2010 treated as current distribution: (i) the prevalence of level 1 and level 2 for each sex-age group was decreased to 0, which means 100% physical activity. This is the same as PAF calculation results, (ii) the prevalence of level 1 and level 2 for each sex-age group was decreased by 50%, and (iii) the prevalence of level 1 and level 2 for each sex-age group was decreased by 30%.

#### Uncertainty analysis

Inevitably, several forms of uncertainty occurred in our estimate, including stochastic variation, systematic biases and errors. It is not reasonable if we just provided a definite point value as the final estimate. Monte Carlo simulation techniques, which consist of assigning probability distributions to model variables that involve risk and then generating random numbers based on those distributions, were used to present uncertainty ranges around point estimates reflecting the main sources of uncertainty. The @RISK software version 6 for Excel [34] allows multiple recalculations of a spreadsheet. We defined the *RR* and risk factor exposure level as the input variables, and the attributable fractions, attributive deaths and LE as the output variables. For the input variables we specified a log-normal distribution for the *RR* and normal distribution for the prevalence of risk factor. For each of the output variables, 95% uncertainty intervals (95% UI) were calculated bounded by the 2.5^th^ and 97.5^th^ percentiles of the 2000 iteration values generated.

## Results

**Table 1** shows the prevalence of three levels of physical activity based on the 2007 and 2010 CDRFSS. These data indicate that level 1 (physical inactivity) and level 2 (low physical activity) were higher in 2010 than 2007, higher for females than males, and increased with age.

**Table 1:** The prevalence of three levels of physical activity in Jiangxi province of China by age-sex, 2007 and 2010.

Year	Age group in years	Level of activity						
		Male			Female			
		Level 1	Level 2	Level 3	Level 1	Level 2		Level 3
**2007**	**18–24**	32.1	34.5	33.4	27.1	49.4		23.5
	**25–29**	37.5	26.3	36.2	38.7	38.1		23.2
	**30–34**	40.9	24.9	34.2	39.1	32.0		28.9
	**35–39**	39.3	26.2	34.5	56.7	26.9		16.4
	**40–44**	43.7	19.9	36.4	52.3	31.6		16.1
	**45–49**	44.1	32.5	23.4	56.2	26.6		17.2
	**50–54**	39.2	33.6	27.2	57.7	28.6		13.7
	**55–59**	42.1	25.3	32.6	66.6	16.9		16.5
	**60–64**	37.3	33.3	29.4	50.1	28.6		21.3
	**65–69**	37.7	35.8	26.5	45.0	35.4		19.6
	**70–74** a	37.7	35.8	26.5	45.0	35.4		19.6
	**75–79** a	37.7	35.8	26.5	45.0	35.4		19.6
	**≧80** a	37.7	35.8	26.5	45.0	35.4		19.6
**2010**	**18–24**	38.4	29.2	32.4	31.1	49.7		19.2
	**25–29**	34.8	33.1	32.1	39.5	42.9		17.6
	**30–34**	44.9	22.7	32.4	54.5	37.2		8.3
	**35–39**	47.9	19.0	33.1	53.1	33.0		13.9
	**40–44**	50.4	25.3	24.3	66.3	23.3		10.4
	**45–49**	45.1	26.0	28.9	66.9	17.6		15.5
	**50–54**	47.3	33.5	19.2		69.3	22.0	8.7
	**55–59**	55.1	28.1	16.8		59.3	21.7	19.0
	**60–64**	50.8	31.1	18.1		66.2	22.7	11.1
	**65–69**	50.1	32.0	17.9		55.4	34.2	10.4
	**70–74** a	50.1	32.0	17.9		55.4	34.2	10.4
	**75–79** a	50.1	32.0	17.9		55.4	34.2	10.4
	**≧80** a	50.1	32.0	17.9		55.4	34.2	10.4

a Due to the limited participants in these age groups, we combined them with age group 65–69 and the same prevalence values were utilized.

The *RR*s and 95% confidence intervals (CI) for selected health outcomes by age are presented in **Table 2**. Except for breast cancer, which included only females, the *RR* values for other health outcomes were similar for both males and females.

**Table 2:** *RR* values (95% CI) for selected health outcomes by age.

Risk level	Age group	Health outcomes^a^ and the *RR* values (95% CI)				
		Ischemic heart disease	Ischemic stroke	Breast cancer^b^	Colon cancer	Type 2 diabetes
**Level 1** c	**18–44**	1.71 (1.58–1.85)	1.53 (1.31–1.79)	1.25 (1.20–1.30)	1.68 (1.55–1.82)	1.45 (1.37–1.54)
	**45–69**	1.71 (1.58–1.85)	1.53 (1.31–1.79)	1.34 (1.29–1.39)	1.68 (1.55–1.82)	1.45 (1.37–1.54)
	**70–79**	1.50 (1.38–1.61)	1.38 (1.18–1.60)	1.25 (1.21–1.30)	1.48 (1.36–1.60)	1.32 (1.25–1.45)
	**≧80**	1.30 (1.21–1.41)	1.24 (1.06–1.33)	1.16 (1.11–1.20)	1.30 (1.20–1.40)	1.20 (1.14–1.28)
**Level 2** c	**18**–**44**	1.44 (1.28–1.62)	1.10 (0.89–1.37)	1.13 (1.04–1.22)	1.18 (1.05–1.33)	1.24 (1.11–1.39)
	**45–69**	1.44 (1.28–1.62)	1.10 (0.89–1.37)	1.13 (1.04–1.22)	1.18 (1.05–1.33)	1.24 (1.11–1.39)
	**70–79**	1.31 (1.17–1.48)	1.08 (0.87–1.33)	1.09 (1.01–1.18)	1.13 (1.01–1.27)	1.18 (1.04–1.32)
	**≧80**	1.20 (1.07–1.35)	1.05 (0.85–1.30)	1.06 (0.98–1.15)	1.08 (0.97–1.22)	1.11 (0.99–1.25)

*RR*, relative ration; CI, confidence interval.

a The 10^th^ revision of international classification of diseases (ICD-10) codes for each health outcomes: Ischemic heart diseases: I20–I25. Ischemic stroke: I63, I65–I67 (except I67.4), I69.3. Breast cancer: C50. Colon cancer: C18. Type 2 diabetes: E11.

b Just for females.

c Level 1: Physical inactivity, defined as doing no or very little physical activity at work, at home, for transport or during discretionary time. Level 2: Low physical activity, defined as doing some physical activity but less than 150 minutes of moderate-intensity physical activity or 60 minutes of vigorous-intensity physical activity a week accumulated across work, home, transport or discretionary domains.

Between 2007 and 2010, the attributable fractions of ischemic heart disease, ischemic stroke, breast cancer, colon cancer, and type 2 diabetes attributable to IPA increased from 23.3% to 25.3%, 15.3% to 17.0%, 16.1% to 16.6%, 20.9% to 23.8%, and 14.7% to 18.5% respectively, with the same trend for both males and females. In total, the PAF of the five outcomes increased from 20.3% in 2007 to 22.4% in 2010 (**Table 3**). Attributable fractions of ischemic heart disease were higher in males than females, and colon cancer and type 2 diabetes were higher in females than males in 2007 and 2010. Attributable fractions were generally higher in the age group 35-69 years, and peaked in the 45-49-year age group for males and 55-59-year age group for females in 2007, and in the 55-59-year age group for males and 50-54-year age group for females in 2010 (data not presented here). Overall, 5 885 (95% UI, 5 047–6 506) deaths, accounting for 2.7% (95% UI, 2.3%–3.8%) of total deaths in Jiangxi province in 2007, and 8 578 (95% UI, 8 227–9 789) deaths, accounting for 3.9% (95% UI, 3.3%–4.5%) of total deaths in 2010, were attributable to IPA. The majority of the attributable deaths were due to ischemic heart disease. The LE gains for elimination of attributable deaths were 0.68 (95% UI, 0.61–0.76) (0.27 for males and 0.41 for females) in 2007, and increased to 0.91 (95% UI, 0.81–1.10) (0.41 for males and 0.50 for females) in 2010 (**Table 3**).

**Table 3:** PAF (%), deaths attributable to IPA and LE gains attributable to IPA reduction by age and sex, Jiangxi province of China, 2007 and 2010.

Year	Health outcomes	Male			Female			Both sexes		
		PAF (%)	Deaths	LE gains	PAF (%)	Deaths	LE gains	PAF (%)	Deaths	LE gains
**2007**	**Ischemic heart disease**	23.5	2 306		23.2	1 779		23.3	4 085	
	**Ischemic stroke**	14.9	705		15.8	590		15.3	1 295	
	**Breast cancer**	–	–		16.1	182		16.1	182	
	**Colon cancer**	19.8	129		22.3	105		20.9	235	
	**Type 2 diabetes**	13.7	55		16.7	34		14.7	89	
	**Total**	20.2	3 196	0.27	20.5	2 690	0.41	20.3	5 885	0.68
	**95% UI**									
	**Lower**		2 751	0.22		2 183	0.38		5 047	0.61
	**Upper**		3 378	0.37		2 809	0.52		6 506	0.76
	**% of total deaths**		2.4			3.3			2.7	
	**95% UI**									
	**Lower**		1.8			2.8			2.3	
	**Upper**		3.3			4.1			3.8	
**2010**	**Ischemic heart disease**	25.9	3 201		24.6	2 673		25.3	5 874	
	**Ischemic stroke**	17.0	977		16.9	839		17.0	1 816	
	**Breast cancer**	–	–		16.6	174		16.6	174	
	**Colon cancer**	23.4	324		24.9	139		23.8	463	
	**Type 2 diabetes**	17.9	157		19.6	94		18.5	251	
	**Total**	22.8	4 659	0.41	21.9	3 919	0.50	22.4	8 578	0.91
	**95% UI**									
	**Lower**		4 426	0.38		3 575	0.44		8 227	0.81
	**Upper**		5 372	0.47		4 709	0.66		9 789	1.10
	**% of total deaths**		3.6			4.4			3.9	
	**95% UI**									
	**Lower**		3.1			3.8			3.3	
	**Upper**		4.3			5.5			4.5	

PAF, population-attributable fractions; LE, life expectancy; IPA, insufficient physical activity; UI, uncertainty interval.

**Figure 1:**
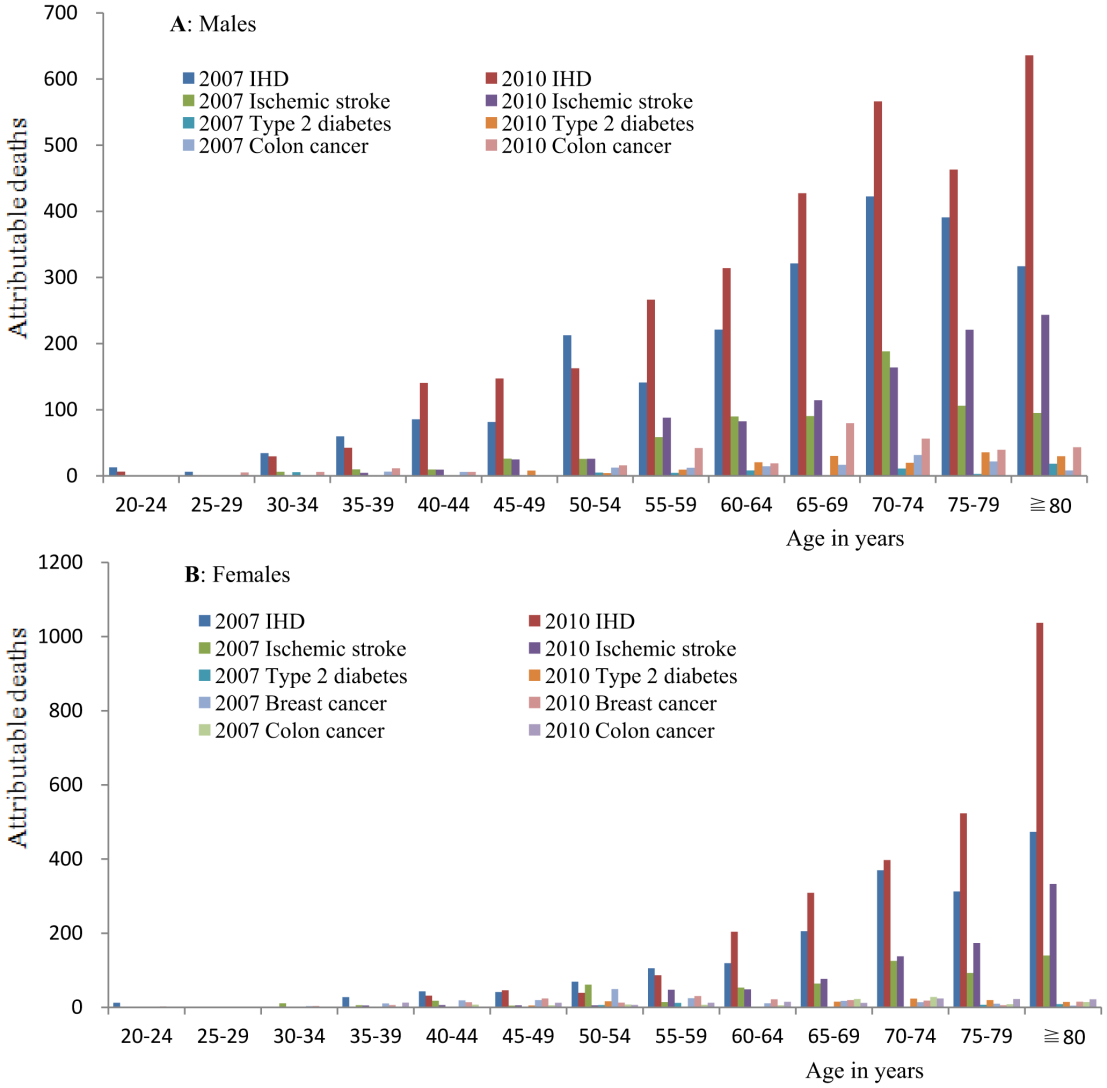
Deaths attributable to insufficient physical activity by age and sex, Jiangxi province of China in 2007 and 2010.

**Figure 1** shows the deaths attributable to IPA across age and sex groups. The overall pattern of deaths for 2010 was different, with a higher proportion at ≧80 years old for females. The total deaths attributable to IPA were higher in older age groups and males and were higher in 2010 than 2007. Most deaths were attributable to ischemic heart disease and ischemic stroke in both 2007 and 2010. A larger proportion of deaths in 2007 occurred in the 50–54 age group, in both males and females, compared to 2010.

**Figure 2:**
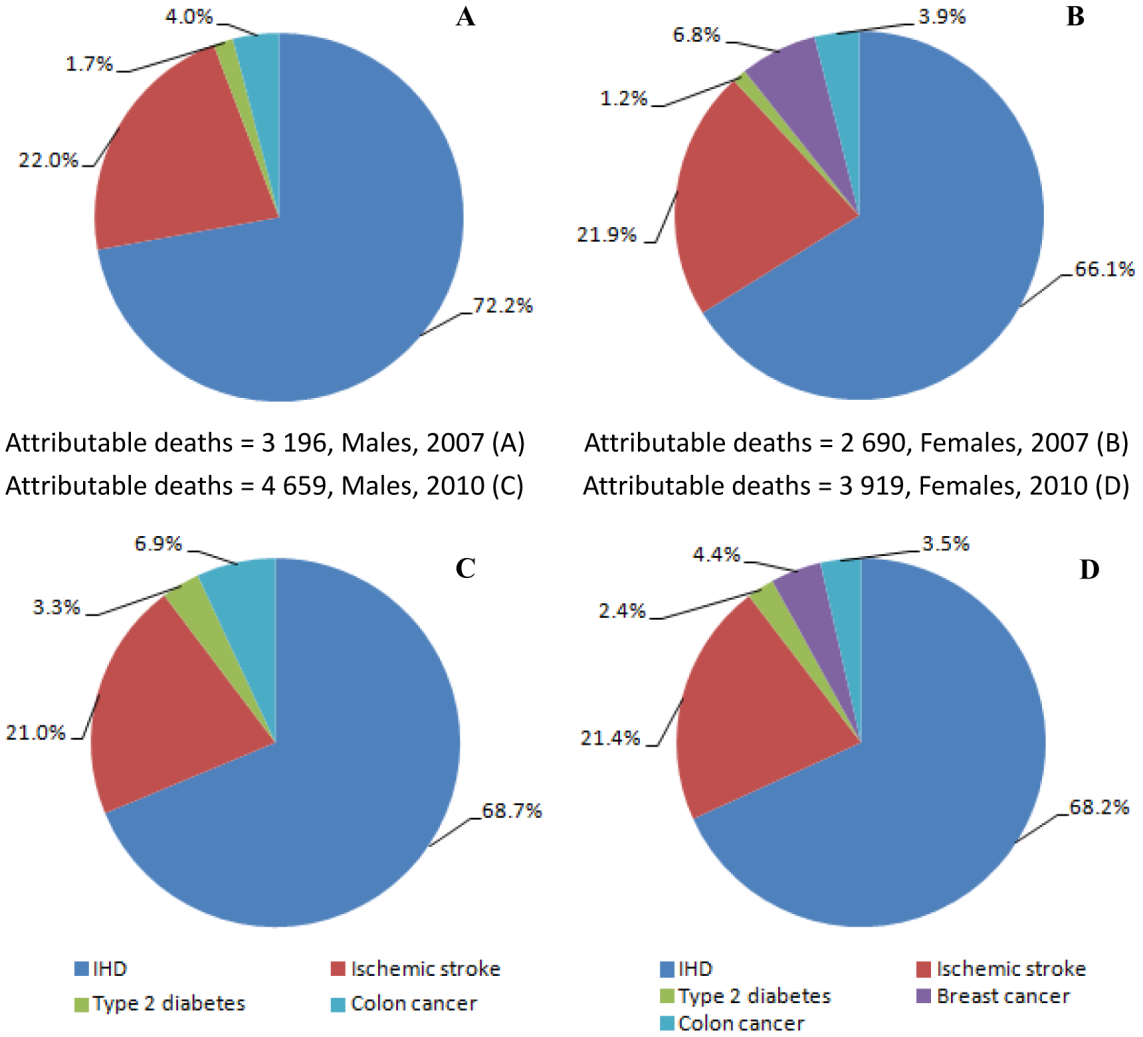
Deaths attributable to insufficient physical activity by sex and conditions, Jiangxi province of China, 2007 and 2010.

**Figure 2** shows that the largest proportion of deaths was caused by ischemic heart disease (more than 65%), followed by ischemic stroke (more than 20%). For females, breast cancer accounted for 6.8% of deaths in 2007 and declined to 4.4% in 2010. Colon cancer accounted for 4.0% in 2007 and increased to 6.9% in 2010 for males. The contribution from type 2 diabetes increased for 2010 among both males and females.

Assuming that the prevalence of level 1 and level 2 for all sex-age groups was decreased by 50% in 2010, 3 678 (95% UI, 3 220–4 229) deaths would have been avoided, with 0.40 (95% UI, 0.34–0.53) years of LE gained (**Table 4**). If the prevalence of level 1 and level 2 for all sex-age groups was decreased by 30% in 2010, 2 090 (95% UI, 1 771–2 533) deaths would have been avoided, with 0.23 (95% UI, 0.16–0.31) years of LE gained (**Table 4**).

**Table 4:** LE shifts at different counterfactual scenarios of IPA by sex comparing with current distribution in 2010, Jiangxi province of China.

Exposure scenarios	Male		Female		Both sexes	
	Deaths	LE gains	Deaths	LE gains	Deaths	LE gains
**Current distribution (i)**	4 659	0.41	3 919	0.50	8 578	0.91
**Decreased by 50% (ii)**	2 668	0.23	2 232	0.28	4 900	0.51
**Decreased by 30% (iii)**	3 529	0.31	2 959	0.37	6 488	0.68
**Burden shifts**						
**(i)–(ii)**	1 991	0.18	1 687	0.22	3 678	0.40
**95% UI**						
**Lower**	1 639	0.11	1 400	0.15	3 220	0.34
**Upper**	2 548	0.24	2 277	0.31	4 229	0.53
**(i)–(iii)**	1 130	0.10	960	0.13	2 090	0.23
**95% UI**						
**Lower**	926	0.05	857	0.08	1 771	0.16
**Upper**	1 417	0.14	1 266	0.17	2 533	0.31

LE, life expectancy; IPA, insufficient physical activity; UI, uncertainty interval.

## Discussion

This study showed an upward trend for the prevalence of IPA, which was associated with higher attributable fractions of deaths from ischemic heart disease, ischemic stroke, breast cancer, colon cancer, and type 2 diabetes from 2007 to 2010 in Jiangxi province of China. Ischemic heart disease and colon cancer accounted for more than 20% of deaths. Although the mortality rate across the country declined from 2007 to 2010, the number of deaths attributable to IPA in 2010 was higher than in 2007. The prevalence of IPA for females was higher than for males. However, the number of attributable deaths for males was greater than for females.

These trends and population distributions of prevalence of IPA in Jiangxi province mirrored the national data [21], [22]. The Global Burden of Disease Study [30] and a 51-country survey [35] also showed that females in most regions of the world were slightly more inactive than males and younger adults were less inactive than older adults. The prevalence of physical inactivity (level 1) in Jiangxi province of China was higher than the global average of 17%, and the prevalence of low physical activity (level 2) was lower than the global average of 40.6% [30]. The findings from the current study suggest that lower levels of physical activity are becoming a global concern.

PAFs for IPA were slightly higher in Jiangxi province than in the global study, in which they accounted for 21.5% of ischemic heart disease, 11% of ischemic stroke, 14% of diabetes, 16% of colon cancer and 10% of breast cancer [30], but lower than those found in other emerging market economies and developed countries [2], [36]. Another study from China that also was based on the 2007 NCDRFSS showed generally lower PAF of ischemic heart disease and type 2 diabetes than our study, but its exposure measure focused only on physical inactivity, with low physical activity excluded [32]. For some important limitations, the global estimates of the 2000 Global Burden of Disease Study were likely to be an underestimate of the true burden attributable to inactive lifestyles [30]. Although the *RR* values used in our study were directly obtained from comparative risk assessment of the Global Burden of Disease Study, it is reasonable to assume that they are valid and robust since the health benefits of physical activity have been demonstrated in a variety of settings and in many developing countries [36]. Physically inactive lifestyles in Jiangxi province were estimated to cause 5 881 and 8 582 deaths, which accounted for 2.7% and 3.9% of total deaths in 2007 and 2010, respectively. Globally, 3.3% of total deaths were due to physical inactivity in 2000 [30] and the same proportion found in South Africa [36]. In summary, our estimates of the morbidity and mortality burden due to physical inactivity in Jiangxi province are plausible and comparable to previous studies. Our findings further support the effort to promote physical activity and reduce inactivity in China.

In this study, we calculated for the first time the LE gains attributable to reducing physical inactivity in China, which showed that elimination of IPA in 2010 would have increased the LE by nearly a year. Any physical activity-related intervention that decreased the prevalence of IPA by 50% and 30% would increase LE by 0.40 years and 0.23 years, respectively. A recent global study in 2008 [37] estimated that elimination of physical inactivity would increase the LE of the world's population by 0.68 years, and in the Chinese population by 0.61 years, which is similar to our study for 2007 (0.68 years).

In addition, we tracked the effects of IPA on mortality and LE in Jiangxi province for different counterfactual exposure distributions, compared with the distribution in 2010. If the prevalence of level 1 and level 2 in each sex-age group was decreased by 50%, 3 678 deaths would have been avoided and 0.40 years of LE gained. If the prevalence declined by 30%, 2 091 deaths would have been avoided and 0.23 years of LE gained. These estimates are expected to be applied to health policy making and evaluation.

To the best of our knowledge, this is the first study to estimate the burden of disease attributable to insufficient physical activity at the provincial level and track the effects on LE in China. However, there were some limitations to our approach. First, the participants were identified using multi-stage stratified random cluster sampling method across the country in each CDRFSS; therefore, the study population might not be provincially representative. Also, the calculation of LE gains was based on two CDRFSSs with cross-sectional designs, which would impact the comparability between 2010 and 2007. The relatively small sample size involved in the 2007 and 2010 surveys [21], [22] might produce unstable prevalence estimates of IPA in Jiangxi province. The same problem exists for the mortality from DSP, which only included 5 points. Fortunately, each field site in CDRFSS is consistent with DSP, which might minimize the impact for the calculation of excess deaths in this study. Lastly, physical activity data were self-reported in CDRFSS, and recall bias and social desirability might influence the exposure estimation [38], [39].

China is going through a rapid demographic change, and unhealthful lifestyles associated with socioeconomic improvement have resulted in a greater burden of non-communicable diseases. Although the Movement of National Healthy Lifestyles initiated by the Ministry of Health and the Nationwide Body-building Plan promoted by the State General Administration of Sport have been implemented for many years [40], sedentary lifestyles in China are still very common. There is an increasing need for the Chinese government to take responsibility for expanding prevention strategies, such as taking action to encourage regular physical activity from childhood to older age, promoting a healthier environment, and motivating non-governmental organizations. Even small reductions in behavioral risk factors could generate substantial health benefits. Preventing or reducing IPA behaviors should be a priority within China's health care reform agenda.
